# Medical Items

**Published:** 1907-05

**Authors:** 


### MEDICAL ITEMS.

### SANMETTO IN PROSTATIC IRRITATIONS, URETHRAL AND BLADDER TROUBLES.

I have used Sanmetto extensively in my practice in prostatic irritation, urethral and bladder troubles, and am well pleased with the results obtained. In cases where the drug is indicated I always feel confident of obtaining good results.—J. A. Downey, M. D., Logansport, Ind.

---

The location of the Head zone will often decide whether a case is one of acute appendicitis with inflammation of the serosa or acute salpingitis. If the Head zone commences at the level of the umbilicus, extends over to the right lumbar region and to just below Poupart's ligament, it is probably acute appendicitis. If the Head zone begins two to three inches below the umbilicus with ? .. cC

---

The Mineral Waters of the Buffalo Lithia Springs have been widely and favorably known for many years. Their efficacy in the treatment and cure of disease has been strikingly attested by their extended use and increasing popularity with each succeeding year, for it has been demonstrated beyond question that their therapeutic action is definite and constant, and that, for special indications they may be relied upon with a great degree of certainty.

Dr. John V. Shoemaker says that Buffalo Lithia Water is doubly efficient in rheumatism and gout. It dissolves uric acid and phosphatic sediments as well as other products difficult of elimination, while at the same time it exerts a moderately stimulant effect upon the renal cells, and thereby facilitates the swift removal of insoluble materials from the body. Without such action insoluble substances will precipitate in the kidneys and bladder. The intense suffering produced by stone, together with consecutive pyelitis and cystitis, are avoided by prompt elimination.

